# A Feasibility Study on Timber Damage Detection Using Piezoceramic-Transducer-Enabled Active Sensing

**DOI:** 10.3390/s18051563

**Published:** 2018-05-15

**Authors:** Jicheng Zhang, Yongshui Huang, Yu Zheng

**Affiliations:** 1 School of Urban Construction, Yangtze University, Jingzhou 434023, China; 100995@yangtzeu.edu.cn (J.Z.); 201672324@yangtzeu.edu.cn (Y.H.); 2 School of Environment and Civil Engineering, Dongguan University of Technology, Dongguan 523808, China

**Keywords:** timber damage detection, piezoelectric transducer, active sensing approach, wavelet-packet-based damage index

## Abstract

In recent years, piezoelectric-based transducers and technologies have made significant progress towards structural health monitoring and damage evaluation for various metal and concrete structures. Timber is still commonly used as a construction material in practical engineering; however, there is a lack of research on the health monitoring of timber-based structures using piezoelectric-based transducers and methods. This paper conducts a feasibility study on timber damage detection using surface-mounted piezoelectric patches, which enable the stress-wave-based active sensing approach. Typical damage modes in timber frame structures, such as surface cracks and holes, were investigated in this study. In the active sensing approach, one piezoceramic transducer is used as an actuator to generate stress waves, which propagate along the surface of the timber structure, and other piezoceramic transducers function as sensors to detect the propagating stress waves. Defects, such as a crack or a hole, induce additional attenuation to the propagating stress wave. Based on this attenuation, the proposed method can detect the defects using the wavelet-packet-based damage index, demonstrating its implementation potential for real-time timber damage detection.

## 1. Introduction

Nowadays, timber is one of the most common construction materials for wood-framed houses [1]. However, timber structures are susceptible to termite attacks, fungal decays, and cracks during their service life due to mechanical loads and environmental effects. It has become increasingly evident that those defects have been major factors in the deterioration of timber structures. These damages in timber structures, if not detected at the earliest possible stage, can weaken their structural loading capacity and shorten their service life. The study of timber damage detection is of great significance to reducing maintenance costs and ensuring structural safety [2].

With the recent rapid advances in structural health monitoring [3,4,5,6] and damage detection technology [7,8], damage detection on timber and wood structures has received increasing attention from researchers over the past decades. Peterson et al. [9,10] and Hu et al. [11] developed statistical algorithms and damage indices by computing the mode shapes of timber beams before and after damage under vibration tests. Subsequently, Choi et al. [12] proposed an improved damage detection algorithm to estimate the status of wooden material by comparing the modal strain energy derived from the first two flexural vibration modes. Similarly, the wavelet transform method [13], the transfer function method [14,15], and the mode shape reconstruction technique [16] were adopted to find the model shapes of timber, which revealed that the mode shapes before and after damage could be further utilized to identify the damage status and location in timber. However, it is difficult to establish real-time monitoring by using such vibration-based methods since external excitations are required in these methods.

Recently, a few real-time methods for timber damage detection have been reported in the literature. Annamdas et al. [17,18] and Wang et al. [19] utilized the piezoelectric impedance method to detect the different types of damage on timber and to evaluate its health condition. Sanabria et al. [20] assessed the bonding quality of glued timbers, and the presence of glue by using the air-coupled ultrasound (ACU) inspection method. Similarly, another ultrasonic testing method was developed by Concu et al. [21] to detect the adhesion status and damage in cross-laminated timber (CLT). By measuring the velocity of ultrasound waves in the wave path perpendicular to the timber plane, the health condition of CLT can be determined. Peterson et al. [22] proposed a combined structural dynamic excitation system and ultrasonic inspection approach to evaluate the integrity of timber bridges. Ross et al. [23] verified the effectiveness of the stress wave method in monitoring a timber bridge by estimating the moisture and decay condition in the wood. In addition, Dackermann et al. [24] proposed a detailed measuring procedure for assessing structural timber by using the stress wave method. By measuring the time of flight (TOF) when the wave propagates across the timber, the moisture condition, decay status, and the effect of annual growth rings can be obtained.

Lead Zirconate Titanate (PZT) is a commonly used piezoceramic material with the advantages of low cost, fast response [25], embeddability [26,27,28,29], strong piezoelectric effect [30], dual ability of actuation and sensing [31,32], wide bandwidth [33], and energy harvesting capacity [34,35]. Due to its wide bandwidth, PZT is often used for stress wave generation [36,37] and detection [38,39,40]. The PZT-enabled active sensing approach using surface-mounted or embedded transducers has shown great potential for the structural health monitoring of mechanical and civil structures in real time [41,42,43,44,45,46]. The principle of this approach is to measure the propagating wave property changes due to the structural damage by using a pair of PZT transducers or a deployed sensor network. An increasing amount of research on structural health monitoring using the active sensing approach has been conducted and reported, including crack and corrosion detection of a metal pipeline system [47,48], monitoring of bolt and rock bolt looseness [49,50,51,52], damage identification in space structures [53], crack and leakage detection of concrete structures [54,55], bond slip detection of composite concrete structures [56,57,58,59], early-age cement hydration monitoring [60,61,62], nondestructive concrete strength evaluation [63], and disbond detection in adhesively bonded structures [64,65]. Moreover, Park et al. [66] proposed a self-powered flexible piezoelectric pulse sensor based on a PZT thin film for a real-time health care monitoring system. Jeonget al. [67] proposed a high-performance (K,Na)NbO3 (KNN)-based flexible piezoelectric energy harvester (f-PEH) using the aerosol deposition method (ADM) with the laser lift-off (LLO) process and conducted experimental tests of cell viability and histological stability to investigate the biocompatibility of both KNN and PZT. However, the study of damage detection methods of timber or timber-supported structures based on the active sensing approach using PZT transducers is still rather limited.

In this paper, a feasibility study on timber damage detection using the PZT-transducer-enabled active sensing approach was investigated. In this investigation is an attempt to employ a stress-wave-based active sensing approach to monitor the cracks and holes in a timber structure. For each timber specimen, one PZT was used as an actuator, which generated guided stress waves to the other PZT that was used as a sensor. Since the stress wave propagation is highly sensitive to the defects on the wave path, timber surface damage such as a crack or a hole can attenuate the stress wave energy. A wavelet-packet-based damage index was applied to characterize the damage severity based on the attenuation ratio of the received wave energy. The analytical results indicated that this damage index had the capability of evaluating crack depths, hole depths, and hole sizes quantitatively and accurately.

## 2. Principles

### 2.1. Active Sensing Approach

**Figure 1:**
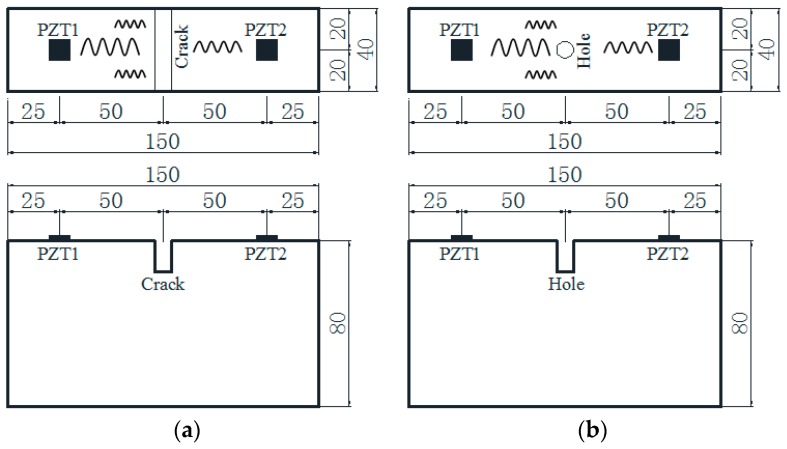
Stress-wave-based active sensing approach for timber damage detection (unit: mm). (**a**) Timber damage with a crack; (**b**) timber damage with a hole.

To monitor time damage using a stress-wave-based active sensing approach, detection equipment was established in this study as shown in Figure 1. Figure 1 demonstrates the principle of this approach to monitor a crack and a hole on a timber beam. It can be seen that a pair of PZT patches (one as an actuator coded as PZT1 and the other as a sensor coded as PZT2) are surface-bonded on a timber specimen to generate and receive stress waves, respectively. When a crack or a hole occurs on the timber surface, the propagating wave energy is reduced by the wave reflection from the defects. This leads to a decreased signal received by the PZT sensors compared to that across an intact timber surface. Additionally, increasing the damage level correspondingly results in a lower received wave energy; damage increase includes increases in crack depth, hole size, and hole depth. In order to quantitatively evaluate the damage severity, a wavelet-packet-based damage index was adopted, which is presented and discussed the next section.

### 2.2. Wavelet-Packet-Based Damage Index

Wavelet packet analysis is a linear time–frequency analysis method with good time–frequency positioning characteristics, which can effectively decompose a variety of time-varying signals. The wavelet-packet-based approach has been widely used in engineering structural analysis [68,69,70,71]. In this investigation, a wavelet-packet-based damage index was used to evaluate the timber damage severity. The procedure of establishing the damage index is as follows:

Firstly, the received signal at the *i*th measurement, $S_{i}$, is decomposed by a 5-level wavelet packet decomposition into $2^{5}$ signal subsets with different frequency bands. The mother wavelet used in this research is “db2”. The signal subset $X_{i,j}$, where *j* is the frequency band (*j* = 1, 2, …, $2^{5}$), can be expressed as

(1) $$X_{i,j}=\{X_{i,j,1},\;X_{i,j,2},\ldots ,X_{i,j,m}\}$$

where *m* is the number of data samplings of the decomposed signal subset.

Secondly, the energy of the signal subset, $E_{i,j}$, can be defined as

(2) $$E_{i,j}=X_{i,j,1}^{2}+X_{i,j,2}^{2}+\ldots X_{i,j,m}^{2}$$

Finally, the damage index *I* is defined using the root-mean-square deviation (RMSD), and it can be given as

(3) $$I\left(i\right)=\sqrt{\frac{\sum_{j=1}^{2^{5}}{\left(E_{i,j}-E_{1,j}\right)}^{2}}{\sum_{j=1}^{2^{n}}E_{1,j}^{2}}}$$

where $E_{1,j}$ of the signal $S_{1}$ refers to the signal that is measured when the structure is healthy. It is clear that the minimum value of the damage index is 0 when there is no damage, as compared with the baseline value, and the maximum value is 1 when the damage is so severe that the sensor receives no signal. It should be noted that a baseline signal response (healthy condition) is always required for this approach. When the baseline signal response (healthy condition) is measured, the RMSD value at the healthy condition is always 0. All the other signal responses measured in damaged conditions are compared to that measured in the healthy condition.

## 3. Experimental Setup

### 3.1. Timber Specimens

**Figure 2:**
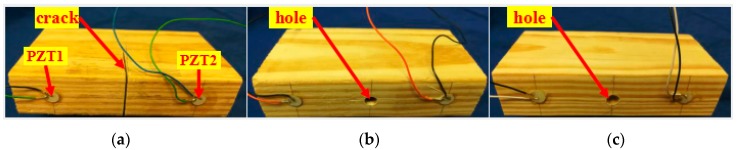
Timber specimens for different groups. (**a**) Specimen of Group A; (**b**) specimen of Group B; (**c**) specimen of Group C.

A total of six timber specimens (pine wood from North America) with the same dimensions were prepared in this experimental test. The dimensions of each test specimen were 0.04 m wide by 0.15 m long and 0.08 m deep. For each specimen, two PZT patches were mounted at the predetermined locations using epoxy (Loctite Heavy Duty 5 min epoxy), as shown in Figure 2. Thus, a thin layer of epoxy exists as the interface between the PZT and the wooden surface. In this research, the PZT sensor was purchased from Beijing Ultrasonic. The PZT sensor is a sandwiched structure with two electrode layers and one layer of PZT material. The dimensions of the PZT sensor are 10 mm in diameter and 0.2 mm in thickness. The six specimens were placed in three groups. It is illustrated in Figure 2 that some artificial structural damages were applied in all the test specimens before monitoring. For Group A (Specimens 1 and 2), a crack was cut on each specimen. For Group B (Specimens 3 and 4) and Group C (Specimens 5 and 6), a hole was drilled in each specimen. The location of the PZT patches and preconfigured damage are presented in Figure 2.

In this study, three different damage types including crack depth, hole depth, and hole size (diameter) were investigated in Groups A, B, and C, respectively (see Table 1). In Group A, cracking damage depths were varied over eight sizes: 0, 2, 4, 6, 8, 10, 20 and 40 mm. The width of the crack was set at 1.5 mm for all test models. For Group B, the effect of hole depth was investigated by varying the structural parameter from 0 to 40 mm over eight steps (see Table 1). The diameter of the hole was fixed at 4 mm for all the models in this group. Finally, five test specimens with different hole diameters were conducted in Group C (see Table 1). This structural variable was varied from 0 to 9 mm to investigate the effect of this structural defect. For the specimens in this group, the depth of the hole was selected to be 4 mm. Table 1 depicts the investigated damage status of all the test specimens in this study.

### 3.2. Experimental Setup and Experimental Procedure

**Figure 3:**
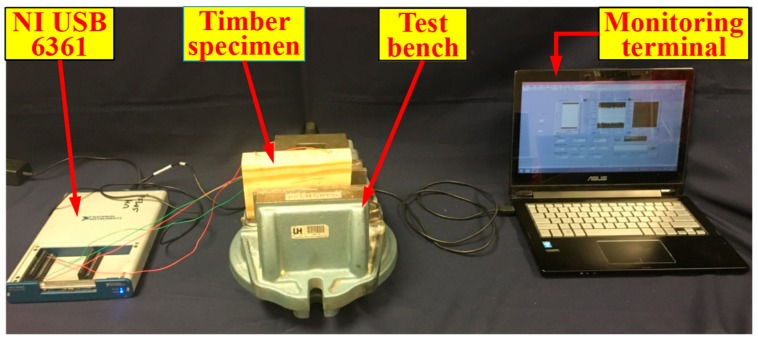
Experimental setup.

Figure 3 shows the experimental setup, including a data acquisition system (NI USB-6361) with a laptop and a timber specimen fixed on a bench. The sampling frequency of the data acquisition system is 2 MS/s. For each case, the PZT actuator was excited by a swept sine wave signal generated guided stress wave from one side of the timber specimen. The PZT sensor recorded the response signal from the other side. The start frequency, stop frequency, amplitude, and period of the excitation signal were 100 Hz, 300 kHz, 10 V, and 1 s, respectively. The frequency step used in this research was 50,000. To minimize the influence of the environmental humidity on the results, all the tests were done within 2 h in the laboratory. The humidity and temperature change within these two hours can be ignored.

## 4. Results and Discussion

**Figure 4:**
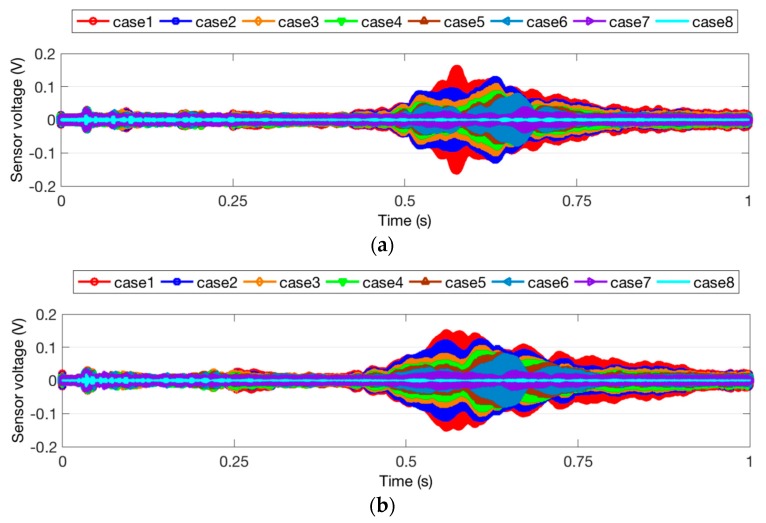
Sensor signal response for Group A. (**a**) Specimen 1; (**b**) Specimen 2.

**Figure 5:**
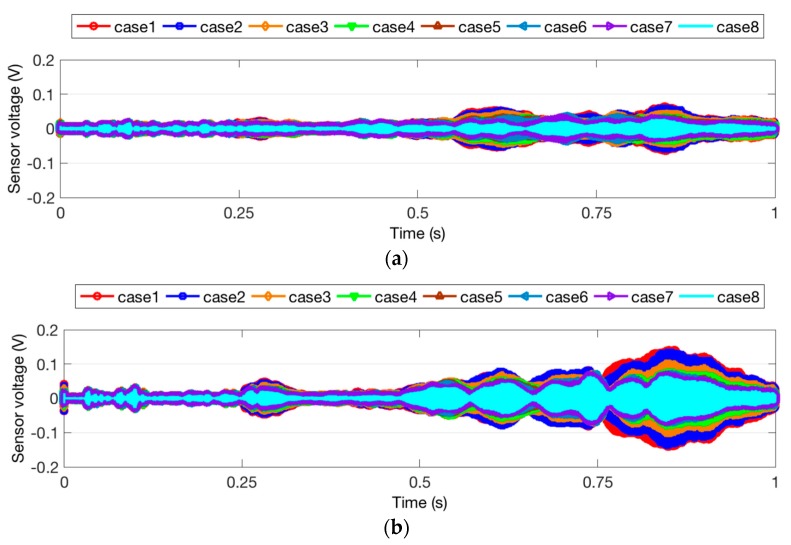
Sensor signal response for Group B. (**a**) Specimen 3; (**b**) Specimen 4.

**Figure 6:**
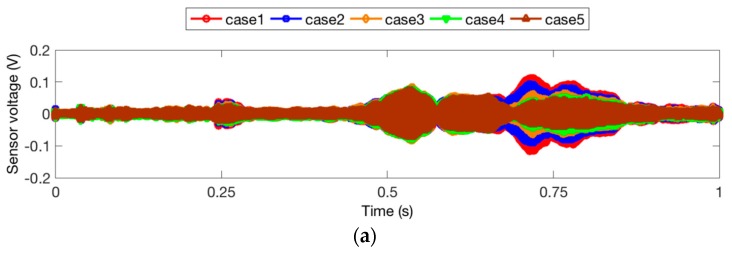

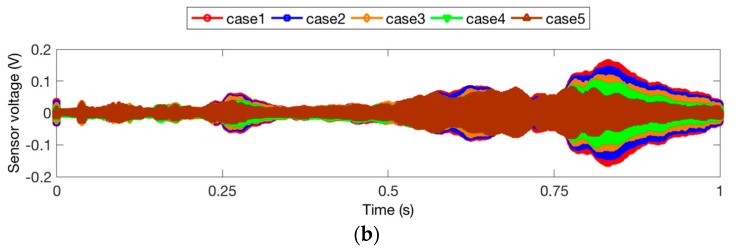
Sensor signal response for Group C. (**a**) Specimen 5; (**b**) Specimen 6.

The time domain signal responses of the PZT sensors of Groups A, B, and C are given in Figure 4, Figure 5 and Figure 6, respectively. Each curve is one period of the sensor signal response of the swept sine wave signal, which is 1 s in the time domain. Due to different damage modes, the received signal of the PZT sensors from different groups is also diverse. In addition, compared to the two specimens with the same damage status in each group, it can be seen that the received signal still presented a unique characteristic. This could be due to the differences among the timber specimens, epoxy dimensions, and PZT wire welding. In the study of a general trend in the signal response of all the test specimens, it was shown that a reduction in the received signal indicated an increase in the timber damage severity. Increasing the cracking depth, hole depth, or hole diameter resulted in significantly smaller stress wave propagation energy on the timber structure. Interestingly, the received signal of Case 8 from Group A is much less than those of Case 8 from Group B and Case 5 from Group C. The reason for this is that deep crack damage across the wave path attenuates much more energy than hole damage.

**Figure 7:**
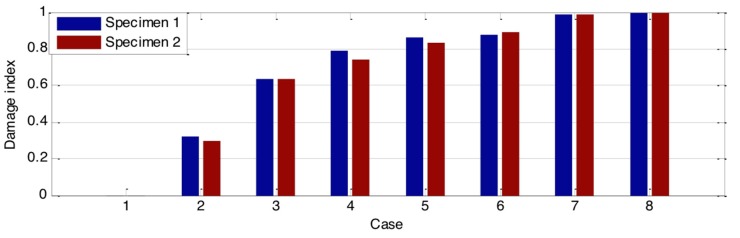
Damage indices of timber with different crack depths.

**Figure 8:**
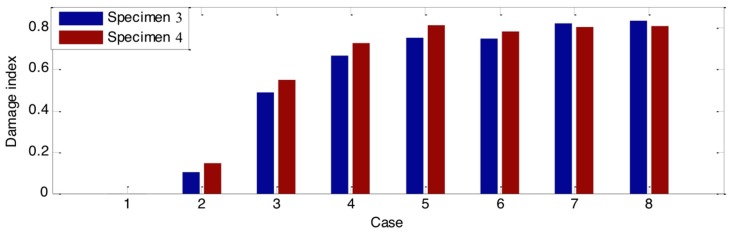
Damage indices of timber with different hole depths.

**Figure 9:**
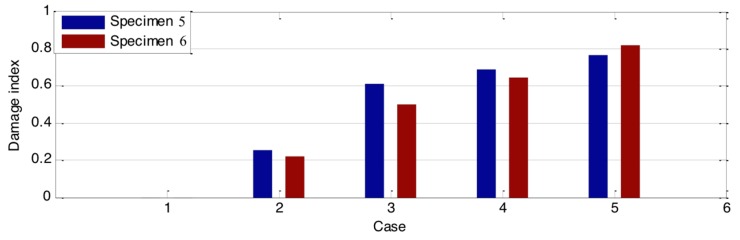
Damage indices of timber with different hole diameters.

Additionally, in order to quantitatively evaluate the loss of the stress wave energy, the wavelet-packet-based energy indices for all the groups were calculated based on Equations (1)–(3). The results of those indices are shown in Figure 7, Figure 8 and Figure 9. Compared to the time domain signal response, those damage indices show promise for estimating the damage severity. In addition, though the time domain signal responses of the two specimens from the same group are different, the values of their damage indices are close to each other. This means that the wave attenuation ratio of the same types of damage is almost the same. As shown in Figure 7 and Figure 8, the damage indices in some cases—Cases 7 and 8 from Group A; Cases 5, 6, 7, and 8 from Group B—reached a constant value, which indicated that the wave attenuation could reach maximum when the crack and hole are at certain depths. In addition, as shown in the damage index (Figure 7, Figure 8 and Figure 9), with the increase of the damage severity, the value of the index correspondingly increases. When the value of the index approaches 1, it means that the structure is subject to severe damage. In the real case, the proposed damage index could provide an early warning for early age damage initiation, as well as a warning when the structure is under severe damage conditions.

The experimental results revealed that the stress-wave-based active sensing approach has great potential to monitor the damage in timber structures. However, there are still many challenges for practical applications using this method. Firstly, the damage mode in timber structures still cannot be identified using these methods, although the structural damage in timber can be detected by the time domain signal responses and wavelet-packet-based damage indices. Secondly, some factors, have not been considered in this research, including humidity, boundary condition, epoxy influences, temperature, wood type, wood size, etc., which could affect the monitoring results. Further research works should be carried in the future to clarify these issues. Finally, another commonly used method called the correlation coefficient deviation metric (CCDM) should be compared with the RMSD method for the structural health monitoring of timber damage.

## 5. Conclusions and Future Work

This research is the first attempt to use a stress-wave-based active sensing approach and a wavelet-packet-based damage index for timber damage monitoring. Two typical types of damage—cracks and holes—were used in this study. The effects of crack depth, hole depth, and hole diameter were investigated by varying those variables. A decreasing trend of the signal amplitude was clearly obtained due to an increase in timber damage. In addition, the damage index values offered a quantitative assessment of the damage severity. The experimental results revealed that this approach could be a promising tool to conduct real-time monitoring of timber damage. To develop further study of this novel monitoring method, the application of a stress-wave-based active sensing approach and a wavelet-packet-based damage index for large-scale timber framing structures with damage will be investigated. In addition, the influences of some variables, such as environmental humidity, boundary condition, epoxy, and temperature, will be considered and investigated comprehensively.

## Figures and Tables

**Table 1:** Test cases of specimens in Groups A, B, C.

Group A	Case	1	2	3	4	5	6	7	8
	Crack depth (mm)	0	2	4	6	8	10	20	40
Group B	Case	1	2	3	4	5	6	7	8
	Hole depth (mm)	0	2	4	6	8	10	20	40
Group C	Case	1	2	3	4	5			
	Hole diameter (mm)	0	3	5	7	9			
